# Editorial: Health information seeking, processing, and sharing

**DOI:** 10.3389/fpsyg.2022.1105612

**Published:** 2022-12-23

**Authors:** Rachel L. Bailey

**Affiliations:** Florida State University, Tallahassee, FL, United States

**Keywords:** health information, health communication, information processing, information seeking, information sharing

Health is important to all aspects of a person's life. Thus, it comes as no surprise that people spend a great deal of time communicating about health. Over the past several decades, digital platforms and technology have made health information widely available, but unfortunately, the credibility of that information is far from consistent. Worse, individuals may have trouble finding and understanding the appropriate information and judging its value and relevance. When erroneous information is shared and spread through social networks, the difficulty of judging the credibility and accuracy of information is amplified.

During the global COVID-19 pandemic, individuals were receiving a great deal of information, not only facts about health but also governmental operations, politics, and the global economy, among many other topics. Along with this valid information surge came a great many rumors, falsehoods, and outright lies. The World Health Organization called this information overload laced with misinformation the “infodemic.”

Fueled by this “infodemic,” health communication research intensified, focusing on how and why people seek the health information they do, how they process it and what factors may influence that processing, what types of behaviors and behavioral intentions can be influenced, and how and why might individuals subsequently share information with others. This Research Topic sought to bring studies from each of these key areas together.

Some overall trends emerged in the submitted studies. Studies examining each of these critical subareas — health information seeking, processing, and sharing alike — are interested in the emotionality and social aspects of information and how those aspects interact with other message factors or individual differences to facilitate health information processes, subsequent attitudes, and behaviors.

In the area of information processes, several studies examine the role of social influences in messaging on behavioral intentions. For example, Wang et al. investigate social nudging information as it contributes to blood donation intentions. Other studies focus on social influences, but in the context of an emotional appeal. Liu et al. examine the combined influence of fear appeals with social norm information on vaccination intentions. Bailey et al. also examine the combination of social information in fear-based messages, finding that social eating cues can helpfully buffer negative responses to fear appeals *via* their positive emotional and motivational aspects, but these aspects may backfire and fail to decrease unhealthy eating intentions.

Other information processing studies in this Research Topic are interested in other types of appeal techniques. Vandeberg et al. examine whether the type of text presentation (narrative vs. expository) influenced vaccination attitudes in individuals of varying vaccination hesitancy. Their findings indicate that motivated processing, rather than narrative persuasion, is a common health persuasion tactic. Myrick et al. also examine the role of different types of appeals in processing and responding to social imagery. They find that in the context of young women receiving sun-safety interventions in a social media context, the types of appeal utilized should consider the type of evoked emotions to create the most promising attentional and attitudinal outcomes.

Other studies are more interested in the individual differences of the information processor, especially individual differences in social and family structures.

For example, Zhang et al. investigate factors that persistently contribute to physical activity intentions, identifying social and family support as critical predictors. Mai et al. examine the role of personality traits in health literacy formation across different family structures. Marschalko et al., on the other hand, were interested in generational differences. They identify different information processing strategies for vaccine-related information across Gen X, Y, and Z Hungarian women, with Gen X and Z focusing more on benefits and Y focusing more on risks.

In the area of information sharing, investigations of affective impacts are again at the forefront. Huang et al. examine how individual experiences of pandemic anxiety influenced whether individuals were more willing to share unverified information that had been previously extensively shared, finding that sharing may be an anxiety coping mechanism in this type of scenario. Further, Li and Wang investigate the role of communication apprehension and health literacy in the willingness to share health information with physicians online and overall patient-physician relationships.

Lastly, in the area of information seeking, perhaps unsurprisingly, bias is a prominent topic. For example, Suzuki and Yamamoto examine the moderating effect of health literacy on confirmation bias in health search selection. Wedderhoff et al. also examine biases, investigating the role of risk feedback in selective exposure to health-related information. Their findings highlight an impetus to select and consume information that would alleviate threats related to the risk raised by messaging.

Another information-seeking study examines information features and their influence on search processes. Wei and Hsu use topic modeling techniques to examine how certain features of online physician profiles expressing their different expertise affected individuals' search processes and responses.

The studies presented here highlight the complex nature of health in the digital age. The information landscape is dense and difficult to navigate given the rising levels of health mis- and disinformation. But the pressing need to advocate for and educate oneself about health and risk is communicated consistently. Thus, the attention given to emotional and social aspects of information, especially in the context of a host of individual differences, including bias, is promising given that misinformation, especially the sort with malicious intent (i.e., disinformation), often capitalizes on emotional appeals and social frames to gain attention and action. A key challenge for health communication research and practice moving forward will be determining how these and other important information characteristics function, especially in certain population subgroups.

## Author contributions

The author confirms being the sole contributor of this work and has approved it for publication.

